# Implications of number-space synesthesia on the automaticity of numerical processing

**DOI:** 10.1016/j.cortex.2012.03.019

**Published:** 2013-05

**Authors:** Limor Gertner, Avishai Henik, Daniel Reznik, Roi Cohen Kadosh

**Affiliations:** a Department of Psychology and the Zlotowski Center for Neuroscience, Ben-Gurion University of the Negev, Beer-Sheva, Israel; b Department of Psychology and the School of Neuroscience, Tel-Aviv University, Tel Aviv, Israel; c Department of Experimental Psychology and the Centre for Functional Magnetic Resonance Imaging of the Brain, University of Oxford, Oxford, England

**Keywords:** Number – space synesthesia, Automaticity, Magnitude processing, Mental number line

## Abstract

Number-space synesthetes visualize numbers in specific spatial configurations. Their spatial-numerical perceptions are assumed to be automatic in nature and have been found to affect performance in various numerical tasks. The current study tested whether synesthetic number-space associations can modulate the well-established *Size Congruency Effect* (SiCE), which is considered to be an indication for the automaticity of numerical processing. Two groups, number-space synesthetes and matched controls, were tested on a numerical Stroop task (Henik and Tzelgov, 1982). In separate blocks, participants were presented with two digits and asked to make comparative judgments regarding either numerical values (numerical comparison) or physical size (physical comparison). Both dimensions were manipulated orthogonally, creating three congruency levels: congruent (e.g., 2 7), incongruent (e.g., 2 7) and neutral (e.g., 2 2 and 2 7 for physical and numerical blocks, respectively). For the numerical block, both synesthetes and controls showed the classic SiCE, indicating similar automatic processing of physical magnitude. However, in the physical block, synesthetes showed a lack of automatic numerical magnitude processing when the numbers to be compared were presented incompatibly with their relative position on the synesthetic number-form. This finding strongly suggests that synesthetes’ number-space perceptions affect their ability to automatically process the semantic meaning of numerals. The involvement of space in automatic magnitude processing for number-space synesthetes and non-synesthetes is discussed.

## 1 Introduction

The interaction between numbers and space was widely established through a myriad of behavioral (e.g., Bachthold et al., 1998; Dehaene et al., 1993; Fisher et al., 2003), imaging (e.g., Cantlon et al., 2009; Göbel et al., 2006; Göbel et al., 2001; Hubbard et al., 2005) and brain damage (e.g., Doricchi et al., 2005; Spalding and Zangwill, 1950) studies. By now, it is well accepted that numerical and spatial representations share common cognitive and neural mechanisms in the human mind and brain (Walsh, 2003).

In recent years, a peculiar condition called number-space synesthesia was recognized to have a great potential for the study of numerical cognition in general and the linkage between numbers and space in particular. Number-space synesthetes are otherwise normal individuals who consciously visualize numbers in specific spatial configurations. In some cases the numbers are arranged in a complex pattern and in other cases they are simply aligned on a horizontal or vertical meridian. These spatial representations seem to be triggered automatically and usually remain constant across a lifetime.

This phenomenon of "visualized numerals" was first introduced in 1880 by Sir Francis Galton (Galton, 1880). However, a century passed before it was experimentally renaissanced. To date, most behavioral research on number-space synesthesia sought to reveal the implicit costs and/or benefits of the synesthetes’ conscious number representation on their numerical cognition (Cohen Kadosh and Gertner, 2011; Cohen Kadosh et al., in press; Simner, 2009; Simner et al., 2009). Specifically, it was found that synesthetes’ spatial-numerical perceptions can affect performance in various numerical tasks, varying from number comparison tasks (Gertner et al., 2009; Hubbard et al., 2009; Piazza et al., 2006; Sagiv et al., 2006; Tang et al., 2008) through parity judgments (Jarick et al., 2009, 2011) up to basic arithmetic exercises (Seron et al., 1992; Ward et al., 2009).

### 1.1 Distance effect and Size Congruency Effect

There are two notable effects in the literature of numerical cognition—the *Distance Effect* (DE) and the *Size Congruency Effect* (SiCE). These two effects demonstrate the fundamental abilities of numerical processing: number representation and processing of magnitude.

The DE was first reported by Moyer and Landauer, 1967. In their study, participants were asked to decide which of two presented digits, ranging from 1 to 9, was numerically larger, and found that reaction time (RT) increased as the numerical distance between digits decreased (e.g., RT for the pair "1 9" was faster than for the pair "1 2"). Since then, this effect was replicated in numerous studies, and considered by many to be an indication for the existence of an implicit mental number line (e.g., Dehaene, 1992; Dehaene and Akhavein, 1995; Restle, 1970; Sekular et al., 1971; Van Opstal et al., 2008).

In a previous study (Gertner et al., 2009) we compared the performance of number-space synesthetes with non-synesthete controls in a standard numerical comparison task. It was found that number-space synesthetes displayed the DE only when the numbers’ locations on a screen matched their relative locations on the specific number form. In contrast, the non-synesthete controls showed the classic DE regardless of the numbers’ orientation and/or position. Based on these results, we suggested that the visuo-spatial, uniquely defined number form interferes with the synesthetes’ ability to represent numbers in a flexible manner. As was stated in previous studies, when number-space synesthetes encounter visual numbers their spatial form ’pops out’ and involuntarily modulates numerical task performance (Hubbard et al., 2009; Piazza et al., 2006; Sagiv et al., 2006).

When the two to-be-compared numbers differ not only in their numerical value but also in their physical size, a SiCE is evidenced. In the classic numerical Stroop task (Henik and Tzelgov, 1982), participants were presented with two digits and were asked to make comparative judgments either regarding the digits’ physical size (physical comparison) or their numerical values (numerical comparison). Both dimensions were manipulated orthogonally, creating three congruency levels: congruent (e.g., 3 5—the numerically smaller number was also physically smaller), incongruent (e.g., 3 5—the numerically smaller number was physically larger) and neutral (e.g., 3 3 in the physical task and 3 5 in the numerical task). The SiCE (i.e., slower RT when dimensions are incongruent than when they are congruent) is a result of the participants’ incapability to ignore the irrelevant dimension. This effect of the task’s irrelevant dimension on performance constitutes an indication for the existence of an automatic process (Cohen Kadosh, 2008; Cohen Kadosh and Henik, 2006; Cohen Kadosh et al., 2007a, 2007b; Cohen Kadosh et al., 2008; Rubinsten et al., 2002; Tzelgov et al., 1992). Accordingly, the appearance of a SiCE strongly suggests that number magnitude, or alternatively physical size, is processed automatically since participants are unable to ignore it even when irrelevant to the task at hand.

The current work aims to examine the affect of number-space synesthesia on the automaticity of numerical processing. We used the size congruity task as we found it to be most suitable for studying unintentional processing (Tzelgov and Ganor-Stern, 2004). To be specific, we employed a numerical Stroop task, similar to the one used by Henik and Tzelgov (1982). In order to extract the synesthetic effects, the design was adjusted in a way that the orientation and location of the presented numbers were manipulated, creating number-line compatible and incompatible conditions. This number-line compatibility was determined with respect to the synesthetes’ number forms. We had two groups of synesthetes; one composed of synesthetes who represent the numbers 1–9 horizontally from left to right and another group that included synesthetes who represent the same numbers vertically from bottom to top.

Table 1 depicts the experimental design in which we controlled the type of comparison (numerical *vs* physical), physical-numerical congruency (congruent, neutral and incongruent) and the number-line compatibility (compatible, incompatible)^1^ for each presentation (horizontal and vertical) separately.

In light of our previous studies (Cohen Kadosh and Henik, 2006; Gertner et al., 2009), we presumed that number-space synesthetes would perform poorly when the number display would not match their number-space associations. Specifically, we anticipated that the SiCE would be affected in the number-line incompatible condition but not in the compatible one. Such a finding in the physical comparison block (i.e., numerical value is irrelevant) would suggest that synesthetes are incapable of automatically processing numerical magnitudes when they are presented incompatibly with their conscious mental representations.

With regard to the controls, we thought it would be interesting to examine how non-synesthetes perform on conditions in which numbers are aligned vertically. Although there is evidence for the existence of a vertical mental number line (e.g., Ito and Hatta, 2004; Schwarz and Keus, 2004), previous experiments suggested that the vertical mode of representation is not the preferable one (Cohen Kadosh et al., 2007a, 2007b; Gertner et al., 2009).

## 2 Method

### 2.1 Participants

Seven number-space synesthetes and a group of 14 non-synesthete controls participated in the study in exchange for a small monetary amount or partial fulfillment of a course requirement. Screening for synesthesia was carried out using a short questionnaire, followed by an open interview. In addition, each synesthete performed a mapping pre-task in which they were required to manually indicate the location of the numbers 1 through 9 on a black computer display.^2^

All synesthetes were right-handed females with a mean age of 24.1 (SD = 3.4) years. Four of them visualize numbers 1–9 horizontally from left to right, and 3 visualize the same numbers vertically from bottom to top. All synesthetes described having forms for several additional sequences (e.g., months, letters, and days of the week) and 3 out of 6 also reported having color associations for a few of these forms.

The control group consisted of undergraduate students who were matched to the synesthetes for gender (all females), age (24.4 years old, SD = .7) handedness (all right-handed) and field of study (social sciences).

All participants were unaware of the experiment’s purpose. They all gave their informed consent and the experiment was approved by local ethics committee.

### 2.2 Stimuli

A stimulus display consisted of two Arabic digits, presented on a computer screen, printed in bold “Arial” font. The digits could appear either to the left and right (horizontal version) or at the top and bottom (vertical version) of the center of a screen, separated by 1 cm. There were 12 possible mixed pairs (1-2, 3-4, 6-7, 8-9, 1-3, 2-4, 6-8, 7-9, 1-6, 2-7, 3-8, 4-9), 8 possible same pairs (1-1, 2-2, 3-3, 4-4, 6-6, 7-7, 8-8, 9-9) and 2 possible font sizes (22 and 30). In line with the classic numerical Stroop task (Henik and Tzelgov, 1982), physical size (i.e., font size) and semantic magnitude (i.e., numerical value) were manipulated orthogonally to create 3 congruency levels: congruent (e.g., 3 5), incongruent (e.g., 3 5) and neutral (e.g., 3 3 and 3 5 for physical and numerical blocks, respectively). In addition, digit spatial location was controlled as well. Thus, each pair could appear compatibly (left-to-right or bottom-to-top) or incompatibly (right-to-left or top-to-bottom) with the numbers’ position on the synesthetic number form.

### 2.3 Procedure

In accordance with the synesthetes’ number forms, there were two versions of the same task: a horizontal one and a vertical one. The synesthetes performed the version that corresponded to their number form, whereas controls performed both versions in two different sessions approximately 2 months apart. The vertical task was always carried out first^3^.Each task consisted of 2 blocks in which participants were asked to make a comparative judgment regarding the numbers’ physical size (physical blocks) and 2 blocks in which they were asked to make a comparative judgment regarding the numbers’ numerical value (numerical blocks). The order of the blocks (2 physical and 2 numerical) was counterbalanced between participants. In each block, pairs of digits (1–9) were presented in a randomized order. Each digit was paired with itself or with a different digit that was numerically larger or smaller (by 1, 2 or 5 units), and appeared twice in 2 different physical sizes (i.e., dimension congruency) and in 2 different spatial locations (i.e., number-line compatibility). An entire block was composed of 144 trials; 48 congruent trials (12 different pairs × 2 different locations on the screen × 2 repetitions), 48 neutral trials and 48 incongruent trials.

A given trial started with a fixation point—a white asterisk at the center of a black screen—for 500 msec. Five hundred msec after the fixation point vanished, a pair of digits appeared and remained visible until the participant responded or for 5,000 msec. The next trial began 1,000 msec after the disappearance of the stimulus.

### 2.4 Apparatus

Data collection and stimuli presentation were controlled by a Compaq computer with an Intel Pentium III central processor. Stimuli were presented on a Compaq S510 monitor. Participants sat approximately 60 cm from the computer screen. A QWERTY keyboard was placed on a table between them and the monitor, and they were asked to respond manually by pressing the key attributed to the numerically larger digit. In the horizontal version, the participants were instructed to press a left key ("F") if the left digit was larger, and to press a right key ("J") if the right digit was larger. In the vertical version, the participants were instructed to press a bottom key ("B") if the bottom digit was larger, and to press a top key ("Y") if the top digit was larger. To avoid a possible artifact in the vertical block, all participants were asked to use their right index finger for the top key and the left index finger for the bottom key.

## 3 Results

Mean RTs of correct responses were calculated for each participant in each condition for the numerical and physical comparisons, separately. These mean values were subjected to 3-way analysis of variance (ANOVA), with physical-numerical congruency (congruent, neutral and incongruent), and number-line compatibility (compatible and incompatible) as within-subject factors and with group (synesthetes and controls) as a between-subject factor. Incorrect, very short (≤150 msec) or very long responses (≥2,000) were excluded from the RT analysis. Mean RTs and ERs (error rates) in the various conditions are presented in Table 2.

### 3.1 Vertical task

The results for the vertical presentation corresponded perfectly with our expectations.

#### 3.1.1 Numerical comparison

**Fig. 1:**
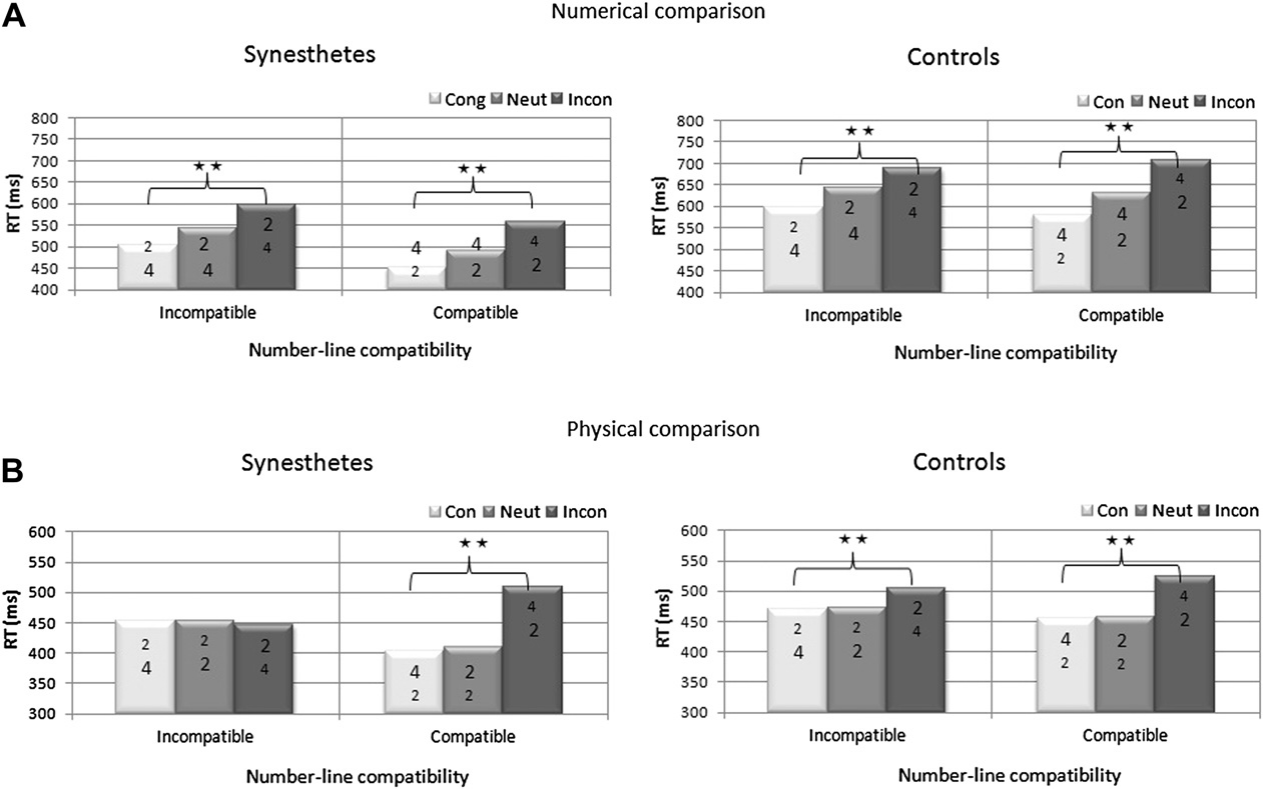
Mean RTs as a function of group, congruency dimensions and number-line compatibility, for numerical judgments (A) and for physical judgments (B) in the vertical task.

A significant main effect was found for dimension congruency [*F* (1, 15) = 57.5, *MSE* = 834, *p* < .0001]. That is, RTs for congruent trials were significantly faster than RTs for the neutral trials, which were significantly faster than RTs for the incongruent trials. Nearly significant effects were found for number-line compatibility [*F* (1, 15) = 4.3, *MSE* = 1882, *p* = .05] as RTs for the compatible condition were faster than RTs for the incompatible condition. No other main effects or interactions were found; meaning the numerical comparison groups did not significantly differ in their patterns of behavior (Fig. 1A).

#### 3.1.2 Physical comparison

A significant main effect was found for dimension congruency [*F* (1, 15) = 19.2, *MSE* = 866, *p* < .0001]. The interaction between congruency and number-line compatibility was found significant as well [*F* (2, 30) = 13.5, *MSE* = 600, *p* < .0001]. Importantly, these two variables also interacted with group [*F* (2, 30) = 4, *MSE* = 600, *p* < .05]. Further analysis of this 3-way interaction revealed that for both synesthetes and controls, congruency and number-line compatibility interacted significantly [*F* (1, 15) = 11.6, *MSE* = 799, *p* < .005; *F* (1, 15) = 4.8, *MSE* = 799, *p* < .05, for synesthetes and controls, respectively], meaning that the congruency effect (RT incongruent – RT congruent) was modulated by the numbers’ position on the screen. Yet, there was a crucial difference between these two interactions. While for controls this interaction was due to a 33 msec larger congruency effect in the number-line compatible condition [*F* (1, 15) = 25, *MSE* = 1,349, *p* < .0005] than in the number-line incompatible one [*F* (1, 15) = 12.7, *MSE* = 732, *p* < .005], for synesthetes this interaction was the result of a significant congruency effect in the number-line compatible condition [*F* (1, 15) = 12.4, *MSE* = 1,349, *p* < .005] with the complete lack of it in the incompatible one [*F* (1, 15) < 1, *ns*] (Fig. 1B).

In order to refute the possibility that this null effect was due to an insufficient statistical power, we conducted a power analysis (one-tailed dependent samples) in which we calculated the optimal sample size required to obtain statistical significance. The power analysis revealed that a sample of 58 participants was needed for this effect to be significant.

#### 3.1.3 ER analysis

We applied the same ANOVA for the ERs as we did for the RTs. The ER results were in line with the RT results. In the numerical comparison, there was a significant effect for dimension congruency [*F* (2, 30) = 23, *MSE* = .002, *p* < .0001] and for group [*F* (1, 15) = 6.2, *MSE* = .003, *p* < .025]. In addition, group interacted with number-line compatibility, meaning that synesthetes had a larger compatibility effect (i.e., more errors for compatibly posited pairs than for incompatibly posited pairs) while the controls did not. However, this interaction was only nearly significant [*F* (1, 15) = 4, *MSE* = .001, *p* = .06]. In the physical comparison, all main effects and interactions were found significant. The most important to our case is the 3-way interaction between congruency, compatibility and group that was found to be significant [*F* (2, 30) = 7.2, *MSE* = .0006, *p* < .005]. Precisely as was found for the RT data, further analysis of the triple interaction revealed that for controls the congruency effect was not modulated by number-line compatibility [*F* (1, 15) = 11.7, *MSE* = .001, *p* < .005*; congruency* *×* *compatibility interaction: F (1, 15)* *<* *1, ns*], while for synesthetes these two variables interacted significantly [*F* (1, 15) = 8.3, *MSE* = .0009, *p* < .025] due to a significant congruency effect in the compatible condition [*F* (1, 15) = 17.2, *MSE* = .001, *p* < .001] but not in the incompatible one [*F* (1, 15) = 2, *MSE* = .0008, *ns*].

### 3.2 Horizontal task

The results for the horizontal task were quite similar although less pronounced than the results for the vertical task.

#### 3.2.1 Numerical comparison

**Fig. 2:**
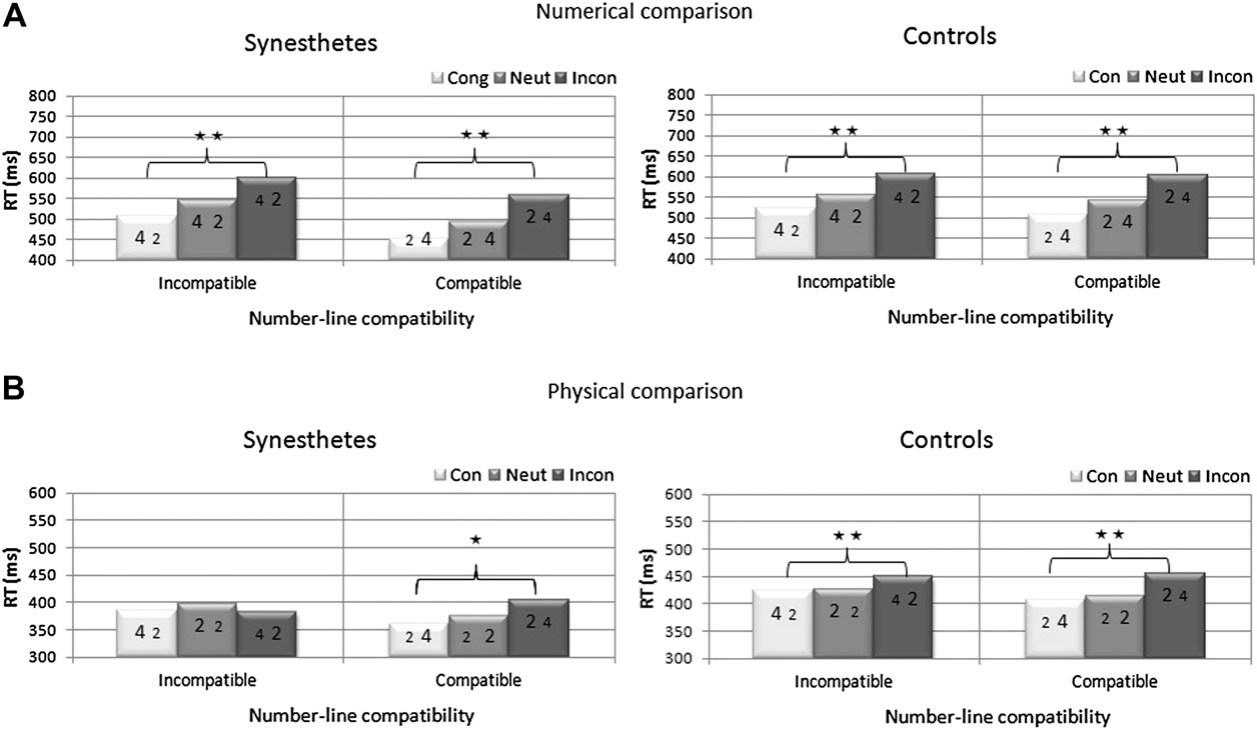
Mean RTs as a function of group, congruency dimensions and number-line compatibility, for numerical judgments (A) and for physical judgments (B) in the horizontal task.

A significant main effect was found for congruency [*F* (2, 32) = 96.3, *MSE* = 583, *p* < .0001] and for number-line compatibility [*F* (1, 16) = 8.2, *MSE* = 1,988, *p* < .025]. The 2-way interaction between number-line compatibility and group was found to be marginally significant [*F* (1, 16) = 3.6, *MSE* = 1988, *p* = .07]. Further analysis revealed a significant number-line compatibility effect (i.e., faster responses to compatibly posited pairs than to incompatibly posited pairs) for synesthetes [*F* (1, 16) = 7.3, *MSE* = 1,988, *p* = .025] but not for controls [*F* (1, 16) = 1, *MSE* = 1,988, *ns*]. Groups did not differ in any other aspect beside this one. No other main effects or interactions were found (Fig. 2A).

#### 3.2.2 Physical comparison

A significant main effect for dimension congruency was found [*F* (2, 32) = 15.2, *MSE* = 366, *p* < .0001] and for number-line compatibility [*F* (1, 16) = 7.3, *MSE* = 148, *p* < .025]. The interaction between congruency and compatibility was found to be significant as well [*F* (2, 32) = 15.2, *MSE* = 143, *p* < .0001]. Unfortunately, this time the triple interaction between congruency, compatibility and group did not reach conventional significance [*F* (2, 32) = 1.9, *MSE* = 143, *p* = .16], nevertheless, with adherence to our predictions, we wished to examine more closely whether the congruency effect was modulated by number-line compatibility differently for each group, and thus we further analyzed this interaction.

As can be infer from the non significant 3-way interaction, both synesthetes and controls displayed a significant 2-way interaction between congruency effect and number line compatibility [*F* (1, 16) = 9.1, *MSE* = 212, *p* < .01; *F* (1, 16) = 8.1, *MSE* = 212, *p* < .025, for synesthetes and controls, respectively]. Further analysis of these interactions revealed a significant congruency effect in both number-line compatibility conditions for the controls, although it was 22 msec smaller for the incompatible condition [*F* (1, 16) = 16.5, *MSE* = 307, *p* < .001] than for the compatible one [*F* (1, 16) = 38.7, *MSE* = 438.3, *p* < .0001]. In contrast, for the synesthetes, a significant congruency effect was evident only in the number-line compatible condition [*F* (1, 16) = 8.2, *MSE* = 438, *p* < .025], but crucially, no congruency effect was found in the number-line incompatible condition [*F* (1, 16) < 1, *ns*] (Fig. 2B).

Again, as before, we conducted a statistical power analysis that revealed a required minimum sample size of 277 participants in order to achieve a significant effect.

#### 3.2.3 ER analysis

In the numerical comparison the only significant effect found was for congruency [*F* (2, 32) = 42.7, *MSE* = .002, *p* < .0001], indicating that both synesthetes and controls displayed a significant congruency effect regardless of number-line compatibility. In the physical comparison, there was a main effect for group [*F* (1, 16) = 7.7, *MSE* = .002, *p* < .025], for congruency [*F* (2, 32) = 28.9, *MSE* = .0005, *p* < .0001] and for number-line compatibility [*F* (1, 16) = 4.9, *MSE* = .0003, *p* < .05]. In addition, number-line compatibility also interacted with group [*F* (1, 16) = 4.9, *MSE* = .0003, *p* < .05]. This interaction was the result of a significant compatibility effect (i.e., more errors for incompatibly posited numbers than for compatibly posited ones) for synesthetes [*F* (1, 16) = 6.3, *MSE* = .0003, *p* < .025] and the lack of it for the controls [*F* (1, 16) < 1, *ns*]. As was the case with the RT data, the 3-way interaction did not reach conventional significance [*F* (1, 16) = 1.9, *MSE* = .0003, *p* = .16].

## 4 Discussion

The current study investigated the influence of number-space synesthesia on simple numerical cognition. Our findings demonstrate that synesthetic number-space associations modulate the automaticity of numerical processing.

First, let us summarize our results. In the numerical comparison, synesthetes and controls displayed a remarkable SiCE, meaning that they were significantly faster to respond to congruent trials than to incongruent trials. The presence of this SiCE was independent of number-line compatibility (i.e., the position of numbers on the screen) and was evident in both horizontal and vertical task versions. In the physical comparison however, the SiCE was modulated by number-line compatibility, for both synesthetes and controls. Yet, there was a crucial difference between the two groups. For the controls, although the SiCE was reduced for the number-line incompatible condition, it was found in both compatibility conditions. However, for the synesthetes, the SiCE was evident only in the number-line compatible condition while it was totally eliminated in the incompatible one. Again, this was the pattern of results for both horizontal and vertical presentations. The ER results coincided with the RT results.

In a classic numerical Stroop task, the processing dimensions (number value or physical size) are manipulated to be relevant or irrelevant to the task at hand. Normal subjects are incapable of ignoring the irrelevant dimension and thus a numerical or physical SiCE is produced (Cohen Kadosh et al., 2008; Henik and Tzelgov, 1982; Rubinsten et al., 2002). This SiCE indicates that the irrelevant dimension was processed irrepressibly and automatically (Cohen Kadosh et al., 2008; Rubinsten et al., 2002; Tzelgov et al., 1992).

In the present study we showed that the numerical SiCE was modulated by synesthetic number-space perceptions. Specifically, in the physical comparison, synesthetes did not show any congruency effect when the numbers were presented incompatibly with their explicit number form. In other words, the synesthetes successfully "managed to ignore" the numbers’ values and thus the numerical SiCE was not produced. This striking finding strongly suggests that synesthetic number-space associations affect the automaticity of processing numerical magnitude.

The numerical SiCE is a fairly robust effect. It was observed in young children (Rubinsten et al., 2002) as well as in elderly individuals (Kaufmann et al., 2008) with or without dementia (Girelli et al., 2001). It was also evidenced in dyscalculic subjects (Rubinsten et al., 2002) and acalculic patients (Ashkenasi et al., 2008) and was even preserved under various non-invasive brain stimulation techniques applied to normal subjects (Cohen Kadosh et al., 2007a, 2007b; Cohen Kadosh et al., 2010). Therefore, it was quite astonishing to discover its total absence in synesthetic individuals.

How can this lack of SiCE be explained? We presume that it might be a matter of shortage in mental resources. In an incompatible condition, the numbers do not match the synesthete’s own conscious representation. This conflict between the mental representation and the concrete visualization necessitates mentally rotating or replacing the numbers’ display to fit their location on the synesthetic number form. This process, which is undoubtedly time and energy consuming, leaves little resources (if any) for processing the numbers’ values. This explanation corroborates previous studies that showed how task difficulty (e.g., perceptual load) can influence performance in general and automatic processing in particular when attentional resources were consumed by high load task (for review see Lavie, 2005; Mattingley et al., 2006). Continuing this line of thought, we suggest two alternatives: One possibility is that synesthetes did perceive the semantic meaning of the numbers to some extent (otherwise there would have been no mistakes at all in this condition), however, the incompatible presentation of the numbers was too difficult for achieving complete automatic processing of the numerical values. Examination of the RT results along with the ER results in physical judgments of the horizontal task support this suggestion, showing that a conflict between the relevant and irrelevant dimensions was evident in the ER measures but did not fully evolve to be manifested also in terms of response time.

Alternatively, it is also possible that when numbers were presented in a "wrong" order, synesthetes did not perceive them as symbols that entailed numerical values but rather as asemantic, meaningless forms. After debriefing, synesthete ES (who has a bottom to top number form) described her insights from the experiment as follows: "*When the numbers are ordered incorrectly, each number stands on its own and is not perceived as a part of the numerical sequence, therefore it is not confusing when the digit does not correspond to the physical size"*.

If this is correct, it would not be farfetched to suggest that for synesthetes, the number-line incompatible condition resembles the neutral condition in the sense that the irrelevant information does not interfere with the relevant information. In the same vein of thought, the congruent condition loses its advantage as a facilitator. Indeed, a closer examination of the facilitation (i.e., neutral RT minus congruent RT) and interference (i.e., incongruent RT minus neutral RT) patterns in the physical block of the vertical task revealed that in the incompatible condition both the interference and facilitation components were eliminated (see Fig. 1B). The ER results for the physical comparison of the vertical task support the above suggestion. For both RT and ER analyses, the SiCE was evident in the number-line compatible condition while it was absent in the number-line incompatible one. This lack of SiCE for both analyses bolsters the assumption that when numbers are presented incompatibly, together with being defined as irrelevant to the task, synesthetes do not perceive them as meaningful symbols that entail semantic information.

Notwithstanding, the above suggestions are valid only when numbers are irrelevant to the task. When numbers were relevant (i.e., the numerical comparison), the SiCE was present regardless of number-line compatibility. Moreover, these SiCEs were not very different in size (92 msec for compatible and 84 msec for incompatible in vertical task; 107 msec for compatible and 94 msec for incompatible in the horizontal task). At first, this finding seemed to deviate from previously reported findings showing that an incompatible presentation of numbers (with respect to the synesthetic number form) affects performance (Gertner et al., 2009; Hubbard et al., 2009; Jarick et al., 2009, 2011; Piazza et al., 2006; Sagiv et al., 2006). However, a closer look at the data revealed that number position did influence general RT. RTs for the number-line compatible condition were significantly shorter than RTs for the number-line incompatible condition in both horizontal and vertical presentations. Moreover, the latter condition was also more prone to errors. Thus, when numbers had to be processed in order to execute the task, as was the case in numerical judgments, synesthetes had to adjust their mental representation to fit the actual one (or vice versa). Although this adjustment slowed down their responses, it did not affect the production of the physical SiCE nor its size.

The current findings converge with our previous data (Gertner et al., 2009) in which we found an elimination of the DE when number-space synesthetes made comparative judgments for digits that were aligned incompatibly with their synesthetic number forms. However, in the previous study, processing numbers were part of the task requirements, that is, they had to be intentionally processed, while in the current study the physical comparison entails an unintentional processing of numbers. These two studies demonstrate the rigidity in the synesthetes’ ability to represent numbers according to task demands. This behavioral inflexibility seems to result in a less effective performance in numerical tasks that require intentional and unintentional numerical processing.

### 4.1 Is number-space synesthesia a magnitude-based phenomenon?

**Fig. 3:**
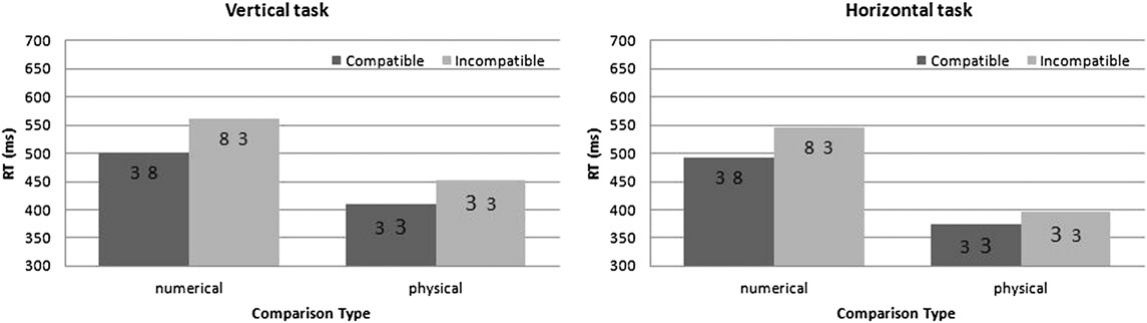
Mean RTs in the neutral condition as a function of number-line compatibility, type of comparison (numerical and physical) and task (vertical and horizontal) for the synesthete participants.

While focusing on the pattern of the SiCE (i.e., incongruent condition RT minus congruent condition RT) we nearly overlooked an interesting pattern regarding the neutral condition itself. A scrutiny of the neutral condition (i.e., one of the dimensions is always held constant) in both comparison types revealed that the spatial position of the numbers modulated response times and accuracy when deciding which number was physically or numerically larger; meaning that synesthetes were faster and more accurate in responding to neutral pairs presented compatibly with their number forms than to neutral pairs presented incompatibly. For numerical judgments this finding is not surprising, and quite expected based on previous research in the field (e.g., Gertner et al., 2009; Hubbard et al., 2009; Piazza et al., 2006; Sagiv et al., 2006). However, for physical judgments (in which numerical value was irrelevant) it was novel and quite amazing to find that physical size solely was affected by spatial position. Specifically, when a large symbol was presented on the left or bottom and a small symbol was presented on the right or top (e.g., 3 3), synesthetes responded significantly less rapidly and less accurately compare to the opposite condition (e.g., 3 3) (Fig. 3, Table 2).

Up to date, number-space synesthesia was viewed as a condition in which spatial concert locations are consciously tied to symbolic numbers (e.g., 2) but not to other non-symbolic quantities (e.g., patterns of dots). However, what if number-space synesthesia is a much wider phenomenon that encompasses not only discrete, ordered, meaningful symbols (i.e., Arabic numbers) but also continuous, non-symbolic magnitudes such as sizes, length, luminance, duration, etc.?

Theories on perception and evaluation of sizes in numerical cognition (for review see Henik et al., 2012) strongly corroborate the above idea, in the sense that an ancient linkage between magnitudes and space exists and perhaps constitutes the neural and cognitive substrates for the evolution of synesthetic number-space associations.

Currently, we are conducting a few experiments in order to test which other aspects of the inducing stimulus might be involved in eliciting a sense of spatial location; is it merely the physical symbol (i.e., Arabic digit), its non-symbolic content (i.e., numerosity/magnitude) or both? We believe such studies will have a significant contribution to the research on number-space synesthesia and to the field of numerical cognition in general.

### 4.2 Non-synesthetic mental number-line

In contrast to the synesthetic explicit mental number form, the implicit numerical representation of non-synesthetes is assumed to be quite pliable and flexible (Bachthold et al., 1998; Cohen Kadosh et al., 2007a, 2007b; Gertner et al., 2009; Schwarz and Keus, 2004). Thus, one does not expect number position to affect the SiCE for control participants. However, our findings show that it does, as was evident by the interaction between dimension congruency and number-line compatibility found in the physical judgments of both horizontal and vertical tasks. These interactions mean that the congruency effects in the number-line compatible condition where more pronounced than the congruency effects in the incompatible condition (see Table 2). That is, when numbers were presented in left-to-right or in bottom-to-top orientations the irrelevant dimension interfered significantly more (and facilitated significantly less) than when the numbers were presented vice versa.

These findings corroborate the idea of *a default preference*. It was previously argued that despite our ability to represent numbers in a flexible manner (compared to synesthetes), we still have a default representation that was established through our daily use of numbers (Cohen Kadosh et al., 2007a, 2007b; Cohen Kadosh and Walsh, 2009; Gertner et al., 2009).

It seems that we generally favor the horizontal orientation over the vertical one, with a controversial tendency to associate small numbers with the left space and large numbers with the right space (Dehaene et al., 1993, but see Wood et al., 2008). However, within the vertical mode, it is well-agreed that the tendency is to associate ’large with top’ and ’small with bottom’ than vice versa (e.g., Gevers et al., 2006; Ito and Hatta, 2004; Rusconi et al., 2006; Schwarz and Keus, 2004). Thus, when the numerical presentations do not correspond to the preferred orientation and the numbers’ semantic meanings are defined as irrelevant to the task, then the numerical magnitude is only roughly processed (or less processed) and a reduction in the size of the congruency effect is observed.

This idea of performing more effectively with one’s preferred orientation applies for both synesthetes and non-synesthetes. Yet, while for non-synesthetes changing the default preference is quite easy and less demanding due to their implicit flexible mental representation, for number-space synesthetes it is far more challenging owing to their conscious, rigid and obligatory number-form.

This is additional empirical data that shows how space constitutes an essential aspect of number representation also in people who do not have an explicit conscious number-line. While the above notions are not entirely new, our study is the first to show that the SiCE can be affected by the spatial presentation of numbers for non-synesthetic controls.

What is the meaning of this in the context of numeral automaticity?

According to the *coalescence model* presented by Schwarz and Ischebeck (2003), one of the factors that explains the SiCE is the level of automaticity of the irrelevant dimension. Specifically, the authors suggest that the greater the automaticity of the irrelevant dimension is, the larger the SiCE will be, and vice versa. Many factors can influence the level of automaticity in numerical processing; for example, the type of notation (Cohen Kadosh et al., 2008), or the familiarity and proficiency of the dimensions at hand (Campbell and Epp, 2004; Henik et al., 2012). We managed to show here that another potential factor that influences the SiCE is space. In our study the spatial location of the numbers affected the strength of their automaticity when they were irrelevant to the task, and the SiCE was modulated accordingly. Specifically, when numbers were presented in a left-right/bottom-top orientation, the level of automaticity was greater than when they were presented in the opposite orientation, therefore the SiCE was reduced in the latter case and increased in the former case.

In our study the spatial location of the numbers affected the strength of their automaticity (when they were irrelevant), resulting in a modulation of the SiCE accordingly.

## 5 Conclusion

The spatial orientation of stimuli affects the processing of those stimuli. We are more accustomed to some presentations, while others are more resource demanding for us. An extreme case is represented by number-space synesthetes, whose conscious, fixed number-space perceptions enabled them to ignore irrelevant numerical values. However, non-synesthetes, who do not possess an explicit number-form and usually display quite a bit of flexibility in their numerical mental representations, also had a preference mode of representation, which affected the processing of the irrelevant numerical dimension.

Our findings further support the idea that both synesthetes and non-synesthetes share the same cognitive mechanisms for associating numbers and space. The observed differences between them lay in the extent to which each group is aware of this number-space interaction. These differences can be further examined under the light of neuronal reuse theories (for review see Anderson, 2010), asserting that brain areas that evolved initially for one cognitive function (e.g., representation of space) reuse these earliest existing structures during evolutionary development to acquire new culturally-driven capabilities (e.g., representation of numbers). If there is a failure in the reuse process (i.e., neural specialization for processing numbers and space), the two functions will stay unspecialized, resulting in a strong, explicit, obligatory association between them. However, if the process is successful, there might still be some indifferently in coding numbers, and space, although to a much lesser extent (Cohen Kadosh and Gertner, 2011). The discussion on reuse theories are beyond the scope of this paper, however we believe that the ideas these theories present might account for the origin of number-space associations in synesthetes and in non-synesthetes, and the commonalities and differences between them.

## Figures and Tables

**Table 1:**
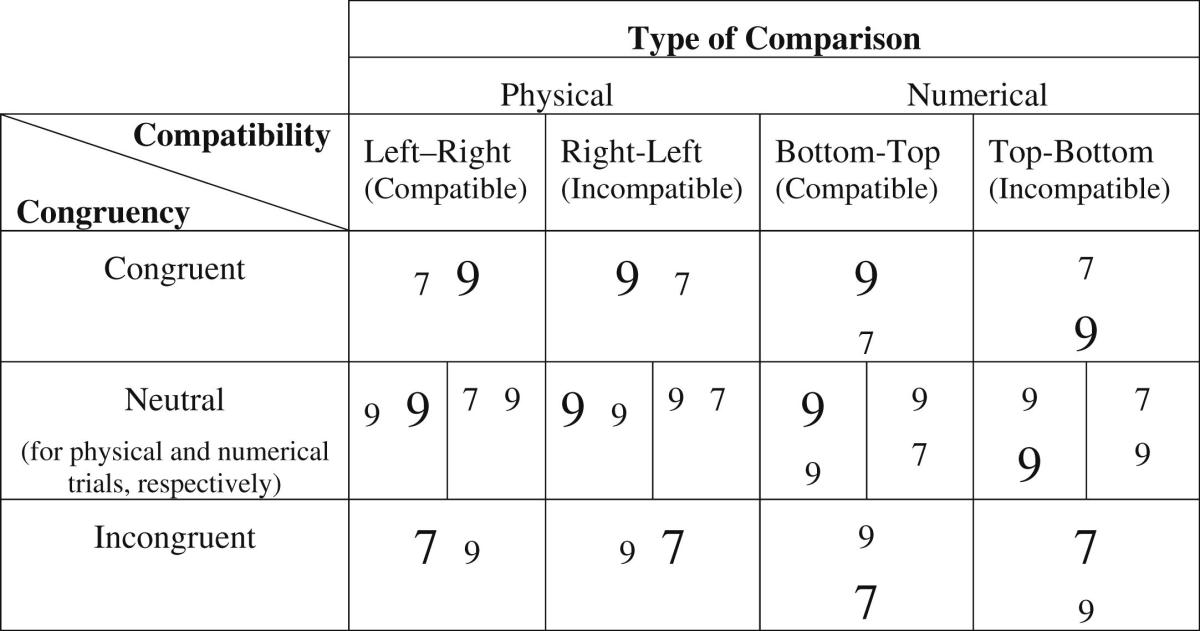
Depiction of the experimental design.

**Table 2:**
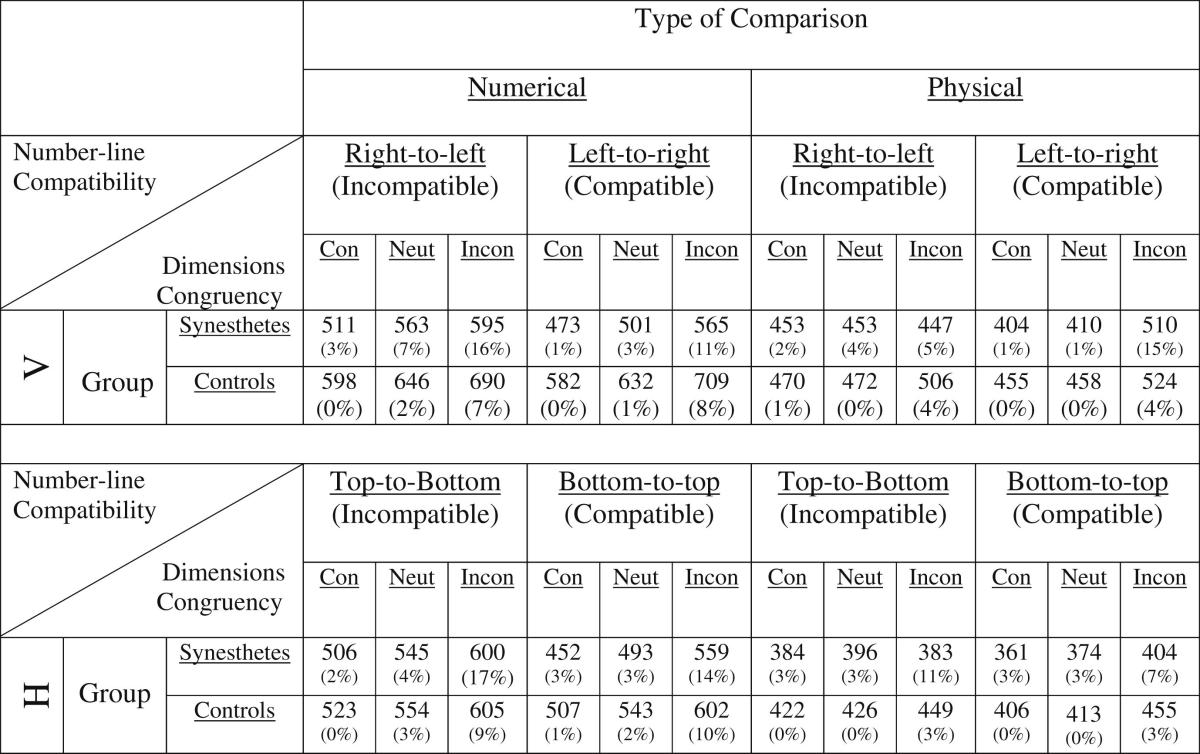
Mean RT (in msec) of Correct Responses and Error Rates (%) in the Various Conditions.

## Footnotes

1 Compatible condition refers to numbers presented in a rightward orientation or upward orientations (i.e., compatible with the synesthetic number-form). Incompatible condition refers to numbers presented in leftward or downward orientations (i.e., incompatible with the synesthetic number-form).

2 In each trial, a white digit between 1 and 9 appeared at the top of a black computer screen. Using the mouse, synesthetes were required to locate the digit on the black display as it appeared in their “mind’s eye”. Each digit was randomly presented for 10 times. X and Y coordinates were recorded.

3 At first we thought to run two separate control groups, one for each task version, arbitrarily starting with the vertical group controls. Later on we decided that one control group would yield more reliable results and recruited the same participants for another session, approximately two months later, in order to perform the horizontal version as well.
